# Tumor Necrosis Factor-*α*-Induced Protein 8-Like 2 Ameliorates Cardiac Hypertrophy by Targeting TLR4 in Macrophages

**DOI:** 10.1155/2022/9469143

**Published:** 2022-04-26

**Authors:** Rui Yao, Lingyao Kong, Chunlei Yang, Jiaqi Du, Guojun Zhao, Yapeng Li

**Affiliations:** Department of Cardiology, The First Affiliated Hospital of Zhengzhou University, Zhengzhou, China

## Abstract

**Background:**

Tumor necrosis factor-*α*-induced protein 8-like 2 (TIPE2), a novel immunoregulatory protein, has been reported to regulate inflammation and apoptosis. The role of TIPE2 in cardiovascular disease, especially cardiac hypertrophy, has not been elucidated. Thus, the aim of the present study was to explore the role of TIPE2 in cardiac hypertrophy.

**Methods:**

Mice were subjected to aortic banding (AB) to induce an adverse hypertrophic model. To overexpress TIPE2, mice were injected with a lentiviral vector expressing TIPE2. Echocardiographic and hemodynamic analyses were used to evaluate cardiac function. Neonatal rat cardiomyocytes (NRCMs) and mouse peritoneal macrophages (MPMs) were isolated and stimulated with angiotensin II. NRCMs and MPM were also cocultured and stimulated with angiotensin II. Cells were transfected with Lenti-TIPE2 to overexpress TIPE2.

**Results:**

TIPE2 expression levels were downregulated in hypertrophic mouse hearts and in macrophages in heart tissue. TIPE2 overexpression attenuated pressure overload-induced cardiac hypertrophy, fibrosis, and cardiac dysfunction. Moreover, we found that TIPE2 overexpression in neonatal cardiomyocytes did not relieve the angiotensin II-induced hypertrophic response in vitro. Furthermore, TIPE2 overexpression downregulated TLR4 and NF-*κ*B signaling in macrophages but not in cardiomyocytes, which led to diminished inflammation in macrophages and consequently reduced the activation of hypertrophic Akt signaling in cardiomyocytes. TLR4 inhibition by TAK-242 did not enhance the antihypertrophic effect of TIPE2 overexpression.

**Conclusions:**

The present study indicated that TIPE2 represses macrophage activation by targeting TLR4, subsequently inhibiting cardiac hypertrophy.

## 1. Introduction

Cardiac hypertrophy is a common pathological process that leads to heart failure when the heart encounters insults, such as hypertension, infarction, valve disease, and cardiomyopathy [1, 2]. In recent decades, studies have found that inflammation from resident macrophages as well as macrophages derived from circulating blood plays an essential role in the transition from cardiac hypertrophy to heart failure [3]. Studies have confirmed that the interaction between macrophages and cardiomyocytes regulates myocardial hypertrophy and survival in experimental models of heart failure caused by pressure overload, hypertension, or acute injury after myocardial infarction [4, 5]. Cardiac macrophages secrete factors associated with inflammation, the extracellular matrix network, hypertrophy, and survival [6, 7]. Thus, a thorough understanding of the regulation of cardiac macrophages and the interaction between macrophages and cardiomyocytes may lead to new targets for the treatment of heart failure.

Tumor necrosis factor-*α*-induced protein 8-like 2 (TIPE2), a novel immunoregulatory protein, has been reported to play an important role in regulating the function of macrophages [8, 9]. Studies have also confirmed the role of TIPE2 in cancer and cell apoptosis [10]. Recently, TIPE2 has been found to act as a negative regulator to protect the heart against myocardial ischemia/reperfusion injury by inhibiting NOD2-induced inflammatory responses [11]. Moreover, Zhao et al. found that TIPE2 is upregulated in T cells in heart tissue in a cardiac allograft model, indicating an association between TIPE2 and cardiac injury [12]. In the present study, we used aortic banding (AB) to establish a cardiac hypertrophy model to elucidate the functional role of TIPE2 in chronic heart insult.

## 2. Methods

### 2.1. Animals

C57BL/6J mice were purchased from the Chinese Academy of Medical Sciences (Beijing, China). Mice were kept in sterile filter cages with a 12 h light/dark cycle, and the environment was maintained at a constant humidity (55 ± 5%) and temperature (23 ± 1°C). Mice had free access to food and water. Mice were randomly divided into the following four groups with 12 mice per group: lentivirus-control (Lenti-NC) + sham group, Lenti-TIPE2 + sham group, Lenti-NC + AB group, and Lenti-TIPE2 + AB group. Mice were injected with Lenti-NC or Lenti-TIPE2 (2 × 10^9^ TU/mL) via the tail vein once every 2.5 weeks. The first injection was 1 week before the AB surgery. For the rescue experiment, mice were injected with TAK-242 (10 mg/kg), a specific TLR4 inhibitor, via intraperitoneal injection twice per week (HY-11109, MCE, NJ, USA). Four weeks after the AB operation, the mice were subjected to echocardiography and hemodynamic measurements and the mice were then sacrificed to collect samples. All animal experiments in the present study were approved by the Institutional Animal Care and Use Committee of Zhengzhou University (Zhengzhou, China).

### 2.2. Aortic Banding Model

Male C57BL/6 J mice (weighing 24–27 g and 8–10 weeks old) were subjected to AB surgery. After anesthetization with 3% sodium pentobarbital, a rodent ventilator was used to maintain mouse respiration. The second and third intercostal tubes were opened, and the aortic arch branch was exposed and ligated using a 26G/27G syringe needle. For mice in the sham group, the same operation was performed without ligation. After closing the chest, the mice were injected with 0.5% bupivacaine to alleviate pain.

### 2.3. Echocardiographic and Hemodynamic Analyses

According to our previous study, 4 weeks after the AB operation, MyLab 30 CV ultrasound (Esoate S.p.A.) and a 10 MHz linear array ultrasound probe were used for echocardiography to evaluate cardiac thickness, ventricular cavity size, and ejection fraction [13, 14]. Hemodynamics were evaluated by cardiac catheterization as previously described [13, 14].

### 2.4. Quantitative Real-Time RT-PCR and Western Blotting

TRIzol was used to extract total RNA from frozen mouse heart tissue and cardiomyocytes. RNA (2 *μ*g) was reverse transcribed into cDNA by oligonucleotide (DT) primers and a Transcription First-Strand cDNA Synthesis Kit (Roche). PCR amplification was performed using SYBR Green PCR Master Mix (Roche) and a LightCycler 480 system (software version 1.5, Roche). The GAPDH gene was used as an internal reference gene to calculate the relative expression.

Frozen heart tissue and cardiomyocytes were lysed with RIPA lysis buffer. Protein samples (50 *μ*g) were separated by SDS-PAGE and then transferred to PVDF membranes (Millipore, Beijing, China). The membranes were incubated with different primary antibodies, including TIPE2, TLR4 (Abcam, 1 : 1000 dilution), P-NF-*κ*B, T-NF-*κ*B, and GAPDH (Cell Signaling Technology, 1 : 1000 dilution). The protein bands were visualized using enhanced chemiluminescence (ECL) reagent (Bio-Rad, Hercules, CA, USA) and imaged using the ChemiDoc MP imaging system (Bio-Rad). The expression of GAPDH protein was used as the internal reference protein.

### 2.5. Histological Analysis

Four weeks after the AB operation, the mouse heart was removed, fixed with 10% paraformaldehyde, embedded in paraffin, and sectioned (5 *μ*M thickness). Hematoxylin and eosin (HE) staining was used to evaluate the area of cardiomyocytes. The collagen deposition fraction was evaluated by picric acid red (PSR) staining. The myocardial cell area (at least 200 per group) and left ventricular collagen fraction were calculated by a quantitative digital image analysis system (Image-Pro Plus, IPP, version 6.0). Heart sections were incubated with anti-CD45 (Abcam, 1 : 100 dilution) or anti-CD68 (Abcam, 1 : 100 dilution) antibodies and then crosslinked with anti-rabbit horseradish peroxidase (HRP) reagent (Gene-tech, Shanghai, China). Finally, the DAB substrate kit (Gene-Tech, Shanghai, China) was used for coloration. A fluorescence microscope was utilized to acquire images and count CD45^+^CD68^+^ cells (10 fields for each heart).

### 2.6. Neonatal Rat Cardiomyocyte (NRCM) Isolation and Culture

NRCMs were isolated and cultured according to our previous study [13]. The hearts of Sprague-Dawley rats aged 1 to 3 days were harvested and cut into small pieces of 1–3 mm^3^. After digestion with trypsin, the samples were collected and filtered. NRCMs were then seeded in 6-well plates (1 × 10^6^ cells/well) with DMEM/F12 containing 10% fetal bovine serum (FBS). To inhibit proliferation of fibroblasts, cells were treated with 0.1 mM 5-bromo-2-deoxyuridine (BrdU, Sigma, B5002). Angiotensin II (Ang II, 1 *μ*M) was used to stimulate NRCMs for 24 hours. Cardiomyocytes were transfected with Lenti-TIPE2 (Vigene, Shandong, China) to overexpress TIPE2 protein.

### 2.7. Mouse Peritoneal Macrophage (MPM) Isolation

Mice were injected intraperitoneally with 3% thioglycolate for 3 days. After sacrificing mice, the abdominal cavity was washed with PBS to collect MPMs. After centrifuging the lavatory solution (300 rpm, 5 min and 4°C), the collected cells were cultured in RPMI 1640 medium containing 10% FBS and 1% penicillin streptomycin. Interferon *γ* (IFN-*γ*) (10 ng/mL, PeproTech) was used to stimulate cells to induce proinflammatory activation [15]. MPMs were transfected with Lenti-TIPE2 (Vigene, Shangdong, China) to overexpress TIPE2 protein.

### 2.8. Coculture of NRCMs and MPMs

MPMs were transfected with either Lenti-NC or Lenti-TIPE2 8 h before IFN-*γ* stimulation. After 24 h of IFN-*γ* stimulation, MPMs were transferred to a transwell and cocultured with NRCMs that were prestimulated with Ang II for 24 h. Twenty-four hours after coculturing, NRCMs were collected for RNA isolation and qPCR analysis.

### 2.9. Detection of Inflammatory Cytokines by ELISA

ELISA kits (BioLegend (430901, 432604 and 431304)) were used to detect tumor necrosis factor-*α* (TNF-*α*), interleukin- (IL-) 1, and IL-6 in mouse hearts, cardiomyocytes, and macrophages. The standard curve was generated using a standard sample, and the corresponding absorbance was measured with an enzyme labeling instrument (Synergy HT, BioTek, United States). The concentration of each sample was then calculated.

### 2.10. Statistical Analysis

All data are represented as the mean ± standard deviation. The differences between groups were analyzed by two-way ANOVA with Tukey's post hoc test. The differences between two groups were analyzed by unpaired Student's *t*-test. A *P* value less than 0.05 indicated statistical significance.

## 3. Results

### 3.1. TIPE2 Is Downregulated in Hypertrophic Hearts

**Figure 1:**
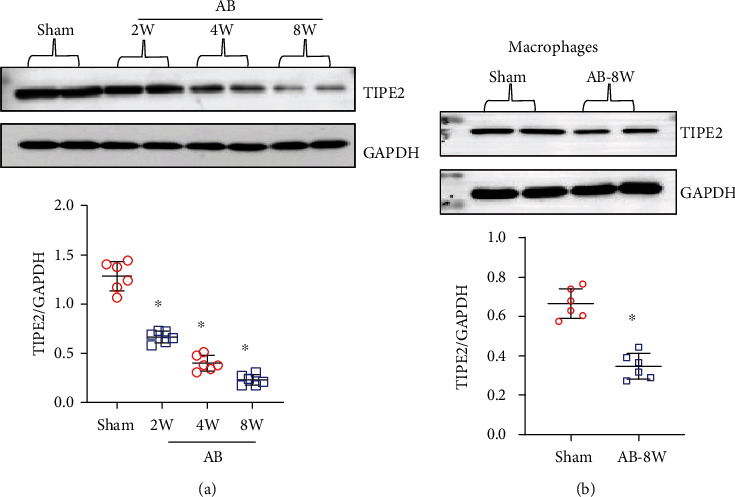
TIPE2 is downregulated in hypertrophic hearts. (a) TIPE2 protein levels in heart tissue post-AB (*n* = 6). (b) TIPE2 protein levels in cardiomyocytes isolated from mice post-AB (*n* = 6). (c) TIPE2 protein levels in fibroblasts isolated from mice post-AB (*n* = 6). (d) TIPE2 protein levels in endothelial cells isolated from mice post-AB (*n* = 6). (e) TIPE2 protein levels in macrophages isolated from mice post-AB (*n* = 6). ^∗^*P* < 0.05 vs. the sham group.

The expression level of TIPE2 was evaluated in mouse hearts at different time points after AB surgery. As shown in Figure 1(a), TIPE2 was downregulated in mouse hearts at 2 weeks until 8 weeks after AB, indicating that TIPE2 participates in the pathological process of adverse hypertrophy. Because heart tissue contains many cell types, we explored which cells have downregulated TIPE2 expression. We isolated cardiomyocytes, fibroblasts, endothelial cells, and macrophages from heart tissue. TIPE2 expression was not detected in cardiomyocytes, fibroblasts, or endothelial cells, but it was downregulated in macrophages (Figure 1(b)). These data suggested that the dynamic changes of TIPE2 in macrophages may affect the progression of adverse hypertrophy to HF.

### 3.2. TIPE2 Overexpression Mitigates Pressure Overload-Induced Cardiac Hypertrophy and Fibrosis

**Figure 2:**
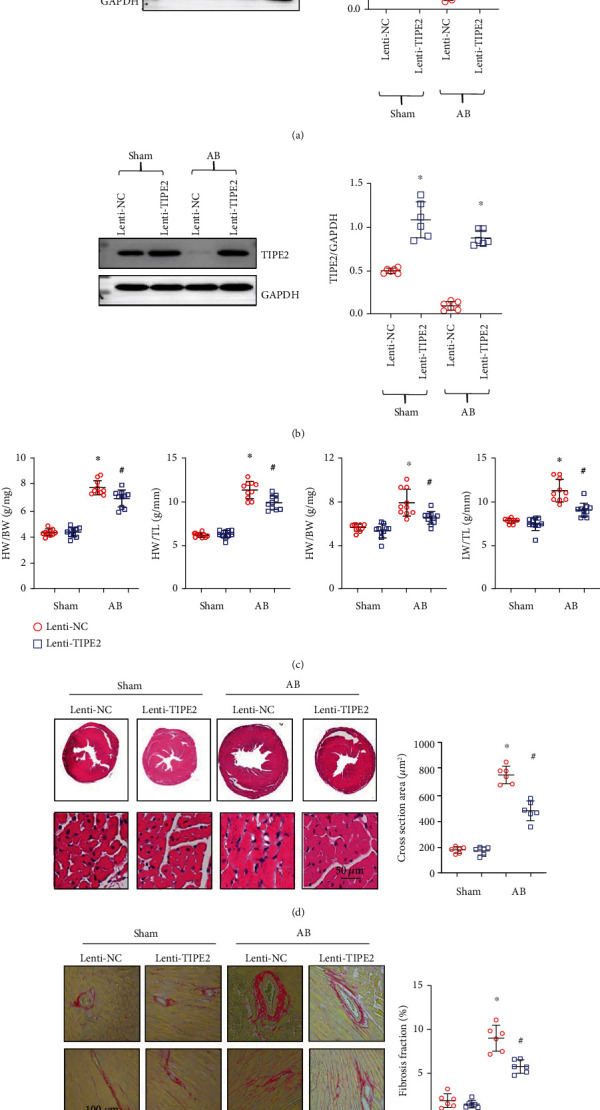
TIPE2 overexpression mitigates pressure overload-induced cardiac hypertrophy and fibrosis. (a) TIPE2 protein levels in hearts from mice injected with Lenti-TIPE2 (*n* = 6). (b) TIPE2 protein levels in macrophages isolated from mouse hearts with Lenti-TIPE2 injection (*n* = 6). (c) Heart weight (HW) to body weight (BW) ratio, HW to tibia length (TL) ratio, lung weight (LW) to BW ratio, and LW to TL ratio (*n* = 10). (d) HE staining and cardiomyocyte cross-sectional area in hearts from mice injected with Lenti-TIPE2 and 4 weeks post-AB (*n* = 6). (e) PSR staining and left ventricular fibrosis area (*n* = 6). ^∗^*P* < 0.05 vs. the Lenti-NC-sham group; ^#^*P* < 0.05 vs. the Lenti-NC-AB group.

We used a lentivirus to overexpress TIPE2 in mice. After 5 weeks of injection, the expression of TIPE2 in heart tissue was markedly increased (Figure 2(a)). We also evaluated the expression of TIPE2 in cardiac macrophages and found that Lenti-TIPE2 injection also induced enhanced TIPE2 expression in macrophages (Figure 2(b)). Four weeks post-AB, the heart weight (HW) to body weight (BW) ratio, HW to tibia length (TL) ratio, lung weight (LW) to BW ratio, and LW to TL ratio were significantly increased in the AB groups (Figure 2(c)). TIPE2 overexpression ameliorated pathological heart growth and lung edema (Figure 2(c)). The gross heart and heart sections were stained by HE (Figure 2(d)). The heart in the lenti-TIPE2-AB group was smaller than that in the control group, and the cross-sectional area of cardiomyocytes was also smaller than that in the control-AB group. Intercellular and perivascular fibrosis, which is another characteristic of cardiac hypertrophy, was also alleviated in the TIPE2-overexpressing group (Figure 2(e)). Thus, TIPE2 may act as a negative factor that inhibits the transition of hypertrophy to HF.

### 3.3. TIPE2 Ameliorates Cardiac Dysfunction

**Figure 3:**
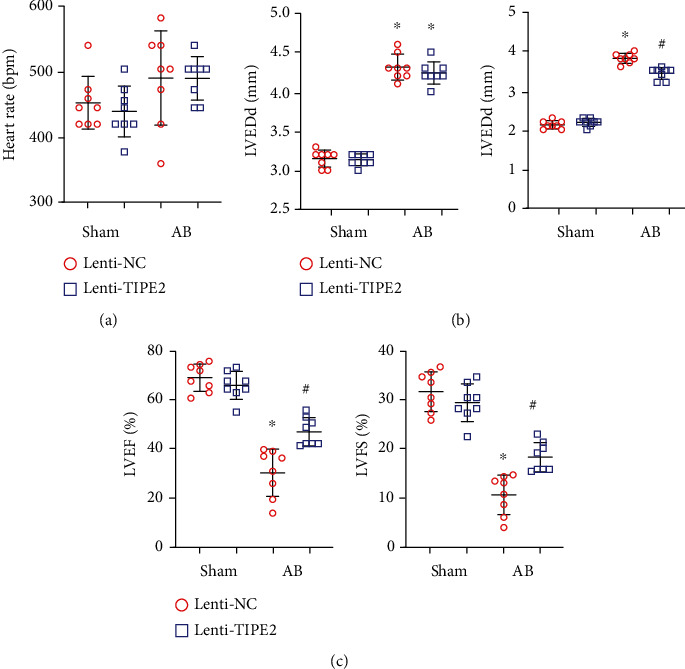
TIPE2 ameliorates cardiac dysfunction. (a–c) Echocardiography of hearts from mice injected with Lenti-TIPE2 4 weeks post-AB (*n* = 8). (a) Heart rate. (b) Left ventricular (LV) end diastolic diameter (LVEDd) and LV end systolic diameter (LVESd). (c) LV ejection fraction (LVEF) and fractional shortening (FS). ^∗^*P* < 0.05 vs. the Lenti-NC-sham group; ^#^*P* < 0.05 vs. the Lenti-NC-AB group.

Echocardiography was performed to evaluate cardiac function. The heart rate showed no difference among the four groups (Figure 3(a)). The left ventricular (LV) end diastolic diameter (LVEDd) was increased in the two AB groups but showed no difference between the two AB groups. The LV end systolic diameter (LVESd) was increased in the AB groups but reduced in the TIPE2-overexpressing group compared to the control-AB group, indicating mitigation of systolic function. The LV ejection fraction (LVEF) and fractional shortening (FS) were improved in the TIPE2-overexpressing group compared to the control-AB group.

### 3.4. TIPE2 Reduces Inflammation in Hypertrophic Hearts

**Figure 4:**
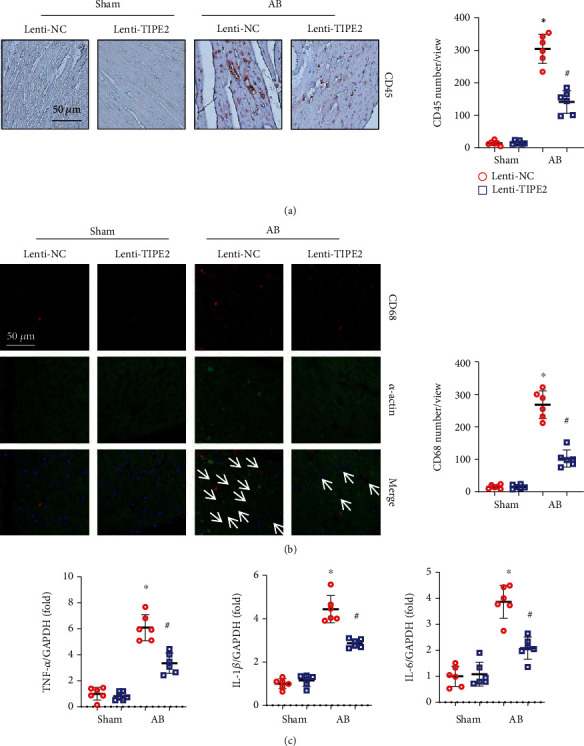
TIPE2 reduces inflammation in hypertrophic hearts. (a) Immunohistochemistry staining of CD45 and quantification of positive cells (*n* = 6). (b) Immunofluorescence staining of CD68 and *α*-actin as well as quantification of positive cells (*n* = 6). (c) mRNA levels of TNF-*α*, IL-1*β*, and IL-6 (*n* = 6). ^∗^*P* < 0.05 vs. the Lenti-NC-sham group; ^#^*P* < 0.05 vs. the Lenti-NC-AB group.

As TIPE2 dynamic change mainly occurred in macrophages, we evaluated the inflammation status in hypertrophic hearts. We used CD45 to label the immune cells and observed that there were fewer CD45-positive cells in the TIPE2-overexpressing group than in the control-AB group (Figure 4(a)). We also used CD68 to label the macrophages in heart tissue and observed that there were fewer CD68-positive macrophages in the TIPE2-overexpressing group than in the control-AB group. Moreover, we assessed the mRNA levels of these proinflammatory factors and found that the transcriptional levels of TNF*α*, IL-1*β*, and IL-6 were reduced in TIPE2-overexpressing hearts (Figure 4(c)).

### 3.5. TIPE2 Does Not Affect Cardiomyocytes

**Figure 5:**
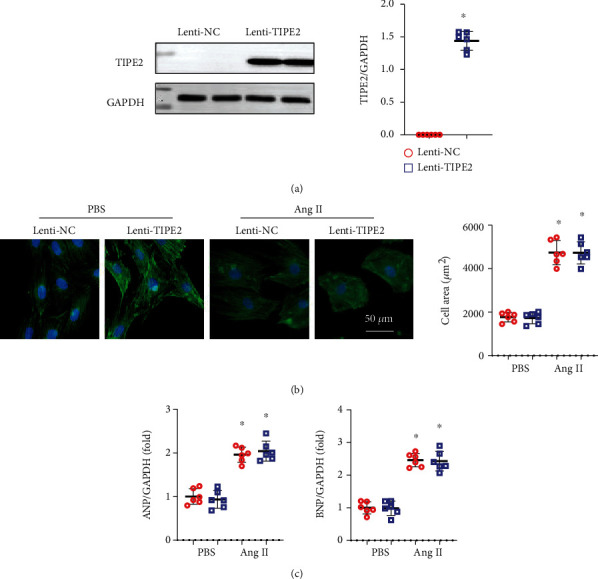
TIPE2 does not affect cardiomyocytes. (a) TIPE2 protein levels in NRCMs infected with Lenti-TIPE2 (*n* = 6). (b) NRCMs were infected with Lenti-TIPE2 and treated with Ang II for 24 h. Immunofluorescence staining of *α*-actin and quantification of the cell surface area (*n* = 6). (c) mRNA levels of ANP and BNP (*n* = 6). ^∗^*P* < 0.05 vs. the Lenti-NC-PBS group; ^#^*P* < 0.05 vs. the Lenti-NC-Ang II group.

To determine whether TIPE2 affects the cardiomyocyte response, we performed in vitro experiments using NRCMs. NRCMs were transfected with Lenti-TIPE2 to overexpress TIPE2 (Figure 5(a)) and stimulated with Ang II to test the hypertrophic response. Ang II induced hypertrophic growth of cardiomyocytes (Figure 5(b)) and increased the levels of atrial natriuretic peptide (ANP) and B-type natriuretic peptide (BNP) (markers of heart failure in response to stress) (Figure 5(c)). However, TIPE2 overexpression did not influence the hypertrophic response with or without Ang II stimulation (Figures 5(b) and 5(c)). These data suggested that cardiomyocytes are not the direct target of TIPE2.

### 3.6. TIPE2 Inhibits Macrophage Proinflammatory Activation

**Figure 6:**
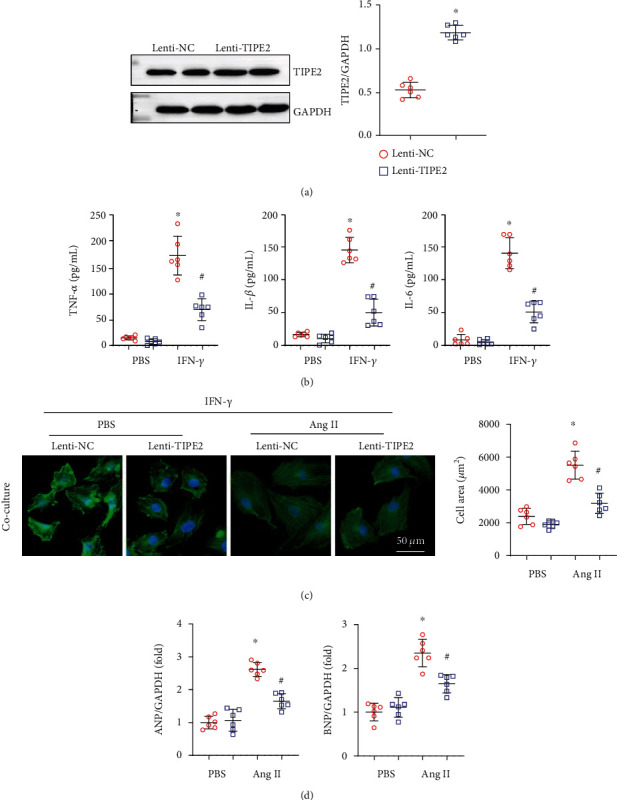
TIPE2 inhibits macrophage proinflammatory activation. (a, b) Macrophages were infected with Lenti-TIPE2 and treated with IFN-*γ* for 24 h. (a) TIPE2 protein levels in macrophages infected with Lenti-TIPE2 (*n* = 6). (b) The levels of TNF-*α*, IL-1*β*, and IL-6 as detected by ELISA (*n* = 6). (c, d) Macrophages were infected with Lenti-TIPE2 and treated with IFN-*γ* for 12 h followed by coculture with NRCMs pretreated with Ang II. (c) Immunofluorescence staining of *α*-actin and quantification of cell surface area (*n* = 6). (d) mRNA levels of ANP and BNP (*n* = 6). ^∗^*P* < 0.05 vs. the Lenti-NC-PBS group; ^#^*P* < 0.05 vs. the Lenti-NC-Ang II group.

As TIPE2 dynamic changes mainly occurred in macrophages and the inflammation status was reduced in TIPE2-overexpressing hearts, we evaluated the influence of TIPE2 in macrophages. MPMs were isolated and treated with IFN-*γ*, and MPMs were also transfected with Lenti-TIPE2 to overexpress TIPE2 (Figure 6(a)). We assessed the proinflammatory factor levels in MPMs and found that TIPE2 overexpression attenuated TNF-*α*, IL-1*β*, and IL-6 levels, consistent with the in vivo results. We then cocultured MPMs (stimulated with IFN-*γ* and transfected with TIPE2) with cardiomyocytes (prestimulated with Ang II). Interestingly, TIPE2-overexpressing MPMs inhibited the Ang II-induced hypertrophic response (decreased cell area and reduced mRNA levels of ANP and BNP) compared to NRMCs cocultured with control MPMs (Figures 6(c) and 6(d)).

### 3.7. TIPE2 Inhibits TLR4 Signaling in Macrophages

**Figure 7:**
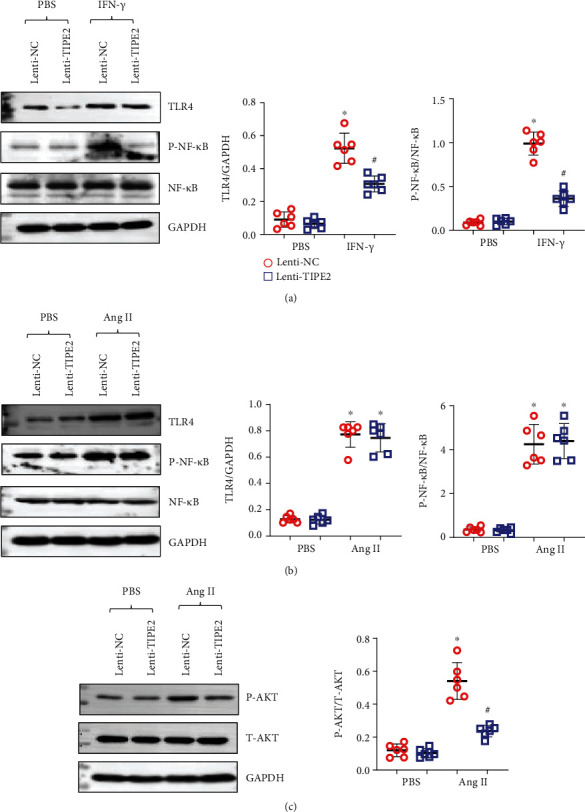
TIPE2 inhibits TLR4 signaling in macrophages. (a) The protein levels of TLR4 and P-NF-*κ*B in macrophages infected with Lenti-TIPE2 and treated with IFN-*γ* (*n* = 6). (b) The protein levels of TLR4 and P-NF-*κ*B in NRCMs infected with Lenti-TIPE2 and treated with Ang II (*n* = 6). (c) The protein levels of P-Akt in NRCMs cocultured with activated macrophages (*n* = 6).

TLR4 is a classic pattern recognition receptor signaling pathway that activates macrophages when activated by damaging molecules or bacteria. We evaluated the protein expression of TLR4 and its downstream molecules. As shown in Figure 7(a), TLR4 and activated NF-*κ*B were increased in activated macrophages, while TIPE2 overexpression reduced the expression of TLR4 and P-NF-*κ*B (Figure 7(a)). We also detected the levels of TLR4 and P-NF-*κ*B in NRCMs overexpressing TIPE2. However, none of these changes were observed in NRCMs. However, in NRCMs cocultured with MPMs, Akt activation was reduced by TIPE2 overexpression in macrophages. Together, these data implied that TIPE2 affects the activation of macrophages in the heart and subsequently regulates the hypertrophic response in cardiomyocytes.

### 3.8. The TAK-242 TLR4 Inhibitor Does Not Affect the Hypertrophic Response in Mice Overexpressing TIPE2

**Figure 8:**
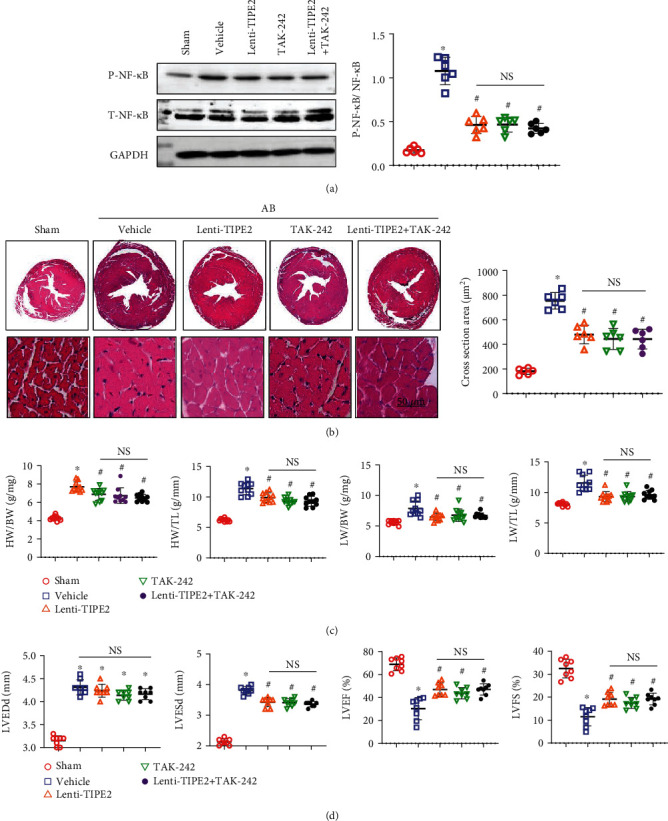
The TAK-242 TLR4 inhibitor does not affect the hypertrophic response in mice overexpressing TIPE2. Mice were injected with the TAK-242 TLR4 inhibitor and/or Lenti-TIPE2. (a) HE staining and cardiomyocyte cross-sectional area of hearts from mice 4 weeks post-AB (*n* = 6). (b) Heart weight (HW) to body weight (BW) ratio, HW to tibia length (TL) ratio, lung weight (LW) to BW ratio, and LW to TL ratio (*n* = 10). (d) Echocardiography of mouse hearts 4 weeks post-AB (*n* = 8). ^∗^*P* < 0.05 vs. the sham group; ^#^*P* < 0.05 vs. the vehicle-AB group.

To confirm whether inhibiting TLR4 further enhances the protective effects of TIPE2 overexpression, mice were treated with TAK-242, which selectively binds to TLR4 and subsequently disrupts the interaction of TLR4 with adaptor molecules. After 4 weeks of TAK-242 injection, downstream P-NF-*κ*B was inhibited in mouse hearts (Figure 8(a)). TAK-242 relieved cardiac hypertrophy in the same manner as TIPE2 overexpression. However, cotreatment of Lenti-TIPE2 and TAK-242 did not act synergistically to further enhance the protective effects against the pathology of HF. The gross heart, cross-sectional area, heart weight, lung weight, and cardiac function showed no difference among the Lenti-TIPE2, TAK-242, and Lenti-TIPE2 + TAK-242 groups (Figures 8(b)–8(d)).

## 4. Discussion

Myocardial injury caused by various factors can trigger the compensatory reaction of cardiomyocytes, cardiac fibroblasts, and inflammatory immune cells, leading to cardiomyocyte hypertrophy, interstitial fibrosis, and heart failure [1]. The present study elucidated for the first time that TIPE2 is downregulated in the progression of cardiac hypertrophy to heart failure and that TIPE2 overexpression inhibits pressure overload-induced cardiac hypertrophy, fibrosis, and dysfunction. Moreover, we found that TIPE2 overexpression in macrophages attenuates inflammatory activation, thereby inhibiting hypertrophic signaling in cardiomyocytes.

During cardiac injury, resident macrophages or those recruited and differentiated from blood-derived monocytes activate and regulate cardiomyocyte homeostasis [16]. In acute cardiac injury, such as myocardial infarction, resident-derived and monocyte-derived macrophages cause inflammation and contribute to fibrosis scar formation [6, 16]. In chronic pathological settings, interactions with macrophages and cardiomyocytes shape the dysfunctional heart [6]. Here, we found that during the transition from cardiac hypertrophy to heart failure, the infiltration of macrophages was increased and proinflammatory factors were also increased. TIPE2 is a negative regulator of immune receptor signal transduction [8], and the normal expression of TIPE2 plays an important role in maintaining immune homeostasis [8]. It has been reported that the expression of TIPE2 is dysregulated in several types of human immune diseases [17]. TIPE2 has been reported to regulate macrophage apoptosis via TLR4 [8] and govern macrophage polarization via mTORC1 [18]. In the present study, we found that TIPE2 was downregulated in macrophages in hypertrophic heart tissue. TIPE2 overexpression mitigated the hypertrophic response and interstitial fibrosis as well as preserved function. This cardioprotective effect of TIPE2 did not rely on the direct function of TIPE2 on cardiomyocytes. We did not observe an influence of TIPE2 overexpression on the hypertrophic response in cardiomyocytes. Instead, TIPE2 reduced the proinflammatory activation of macrophages. Thus, less-activated macrophages may suppress the hypertrophic response of cardiomyocytes to Ang II.

The proinflammatory factors released mainly by macrophages include TNF-*α*, IL-1*β*, and IL-6, and studies have confirmed the prohypertrophic role of these factors [19–21]. Crosstalk between Ang II and TNF has been demonstrated by TNF knockout. TNF knockout attenuates Ang II-induced hypertension by reducing the expression of angiotensin-type 1 receptor [19]. An anti-IL-1*β* antibody can rescue cardiac hypertrophy after transverse aortic constriction [20]. IL-6 signaling inhibits basal contractility and the *β*-adrenergic response in cardiomyocytes [21]. In the present study, TIPE2 overexpression inhibited the release of TNF-*α*, IL-1*β*, and IL-6 from macrophages, and when these macrophages were cocultured with cardiomyocytes, the hypertrophy response to Ang II was attenuated.

TLR4 is a member of the pattern recognition receptor (PRR) protein family, which recognizes pathogen-associated pattern molecular (PAPM) and damage-associated pattern molecular (DAPM) [22]. During chronic cardiac injury, TLR4 is activated by DAMPs and then conveys the activating signal through myeloid differentiation protein 88 (MyD88) and NF-*κ*B [23]. The activation of NF-*κ*B promotes the transcription of several different inflammatory cytokines and chemokines [23]. Previously, TIPE2 has been reported to affect TLR4 signaling in macrophages [8, 11]. Consistently, we found that TIPE2 decreased TLR4 expression and inhibited NF-*κ*B activation in macrophages. Similar to the effect of TIPE2 overexpression, treatment with a TLR4 inhibitor mitigated the adverse cardiac hypertrophy. Moreover, combined treatment with TIPE2 overexpression and a TLR4 inhibitor did not enhance the antihypertrophic effects. These results indicated that TLR4 in macrophages but not in cardiomyocytes is the target of TIPE2. In the present study, we also found a reduction in Akt signaling in cardiomyocytes. As a classic hypertrophic signaling pathway, Akt is activated by Ang II and *β*-adrenaline [24, 25]. During the pressure overload hypertrophy model, both the sympathetic system and RAAS are activated, which triggers the activation of Akt signaling [26]. The reduced inflammation driven by TIPE2 overexpression may lead to limited collaboration of inflammatory factors with Ang II and *β*-adrenaline.

In summary, TIPE2 protects against adverse cardiac hypertrophy by targeting TLR4 in the membrane of macrophages in the heart and subsequently attenuating prohypertrophic Akt signaling in cardiomyocytes.

## Data Availability

All data that support the findings in this study are available from the corresponding author upon reasonable request.
